# Assembly and disassembly of branched ubiquitin chains

**DOI:** 10.3389/fmolb.2023.1197272

**Published:** 2023-06-01

**Authors:** Justin B. Gregor, Dantong Xu, Michael E. French

**Affiliations:** ^1^ Department of Biochemistry, Purdue University, West Lafayette, IN, United States; ^2^ Department of Chemistry and Biochemistry, Middlebury College, Middlebury, VT, United States; ^3^ Department of Chemistry and Biochemistry, University of Tampa, Tampa, FL, United States

**Keywords:** ubiquitin, ubiquitylation (ubiquitination), branched ubiquitin chain, protein degradation, proteasome

## Abstract

Protein ubiquitylation is an essential post-translational modification that regulates nearly all aspects of eukaryotic cell biology. A diverse collection of ubiquitylation signals, including an extensive repertoire of polymeric ubiquitin chains, leads to a range of different functional outcomes for the target protein. Recent studies have shown that ubiquitin chains can be branched and that branched chains have a direct impact on the stability or the activity of the target proteins they are attached to. In this mini review, we discuss the mechanisms that control the assembly and disassembly of branched chains by the enzymes of the ubiquitylation and deubiquitylation machinery. Existing knowledge regarding the activities of chain branching ubiquitin ligases and the deubiquitylases responsible for cleaving branched chains is summarized. We also highlight new findings concerning the formation of branched chains in response to small molecules that induce the degradation of otherwise stable proteins and examine the selective debranching of heterotypic chains by the proteasome-bound deubiquitylase UCH37.

## Introduction

Ubiquitin is a versatile post-translational signal that can impact the stability, activity, localization, and interaction properties of protein substrates in numerous ways. It is most commonly attached to lysine residues of proteins through an isopeptide bond, although a number of other conjugation sites and non-protein targets have been reported (Squair and Virdee, 2022; Dikic and Schulman, 2023). The versatile nature of ubiquitin as a modifier stems from its capacity to be incorporated into a number of distinct structures. Ubiquitin can be conjugated to substrates as a monomer on one or more sites, a modification referred to as monoubiquitylation or multi-monoubiquitylation, respectively. Alternatively, it can be polymerized to form a chain, in which the carboxy terminus of one ubiquitin (the donor) is linked to another ubiquitin (the acceptor), most commonly through an isopeptide bond at one of the eight amino groups on the surface of ubiquitin. At least 12 different sites of chain formation have been reported to date, including several serine and threonine residues (Swatek and Komander, 2016; Kelsall et al., 2019; Rodriguez Carvajal et al., 2021), resulting in a staggering number of potentially unique chain architectures that can be formed when variation in chain length and topology are taken into account.

**FIGURE 1:**
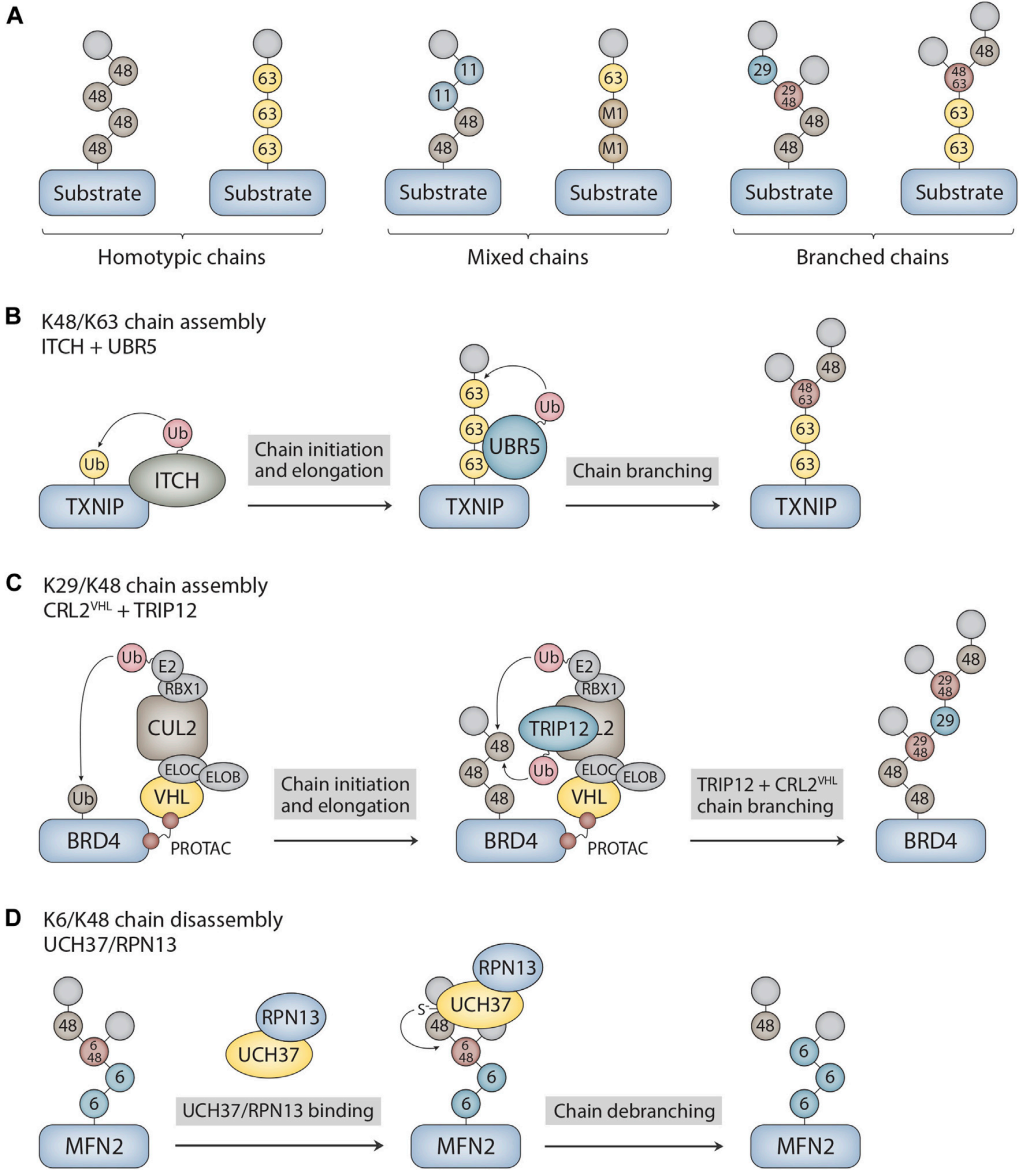
Ubiquitin chain architectures and mechanisms of branched chain assembly and disassembly. **(A)** Ubiquitin chains can be classified as either homotypic or heterotypic (mixed and branched) based on the types of ubiquitin linkages and how the subunits are connected to each other within the chain. Examples of homotypic K48- and K63-linked chains are shown. Other sites of chain formation include M1, K6, K11, T12, S20, T22, K27, K29, K33, and T55. Mixed K11/K48 and M1/K63 chains as well as branched K29/K48 and K48/K63 chains are shown as examples of heterotypic chains. The modified acceptor sites of the ubiquitin subunits are indicated in the figure. The terminal (unmodified) subunits are colored in light grey. **(B)** Mechanism of branched chain formation by the HECT ubiquitin ligases ITCH and UBR5. ITCH attaches homotypic K63-linked chains to TXNIP. UBR5 then binds to the K63 linkages through its UBA domain to nucleate the formation of K48 linkages, resulting in the assembly of branched K48/K63 chains. **(C)** PROTAC-dependent formation of branched chains by CRL2^VHL^ and TRIP12. CRL2^VHL^ assembles homotypic K48-linked chains on BRD4 and then recruits TRIP12, which attaches K29 linkages and further stimulates CRL2^VHL^ activity. This cascade leads to the formation of complex branched K29/K48 chain architectures on BRD4. CLR2^VHL^ subunits include VHL, elongin B (ELOB), elongin C (ELOC), CUL2, RBX1, and E2. **(D)** Mechanism of branched chain disassembly by the proteasome-bound UCH37/RPN13 complex. UCH37/RPN13 binds to both distal ubiquitin subunits that emanate from the K6/K48 branch point. Cleavage of the K48 linkage at the branch point is greatly stimulated by RPN13, resulting in trimming of the chain at the proximal K48 linkage and the release of homotypic K6- and K48-linked chains. The K6-linked chain attached to MFN2 is then presumably cleaved *en bloc* by RPN11, an essential proteasome-associated DUB component of the 19S regulatory particle, prior to MFN2 degradation. MFN2 was identified as a substrate of UCH37 in Du et al. (2022).

Ubiquitin chains can be classified into two different general categories based on the types of ubiquitin linkages and the topology of the chain (Figure 1A). Homotypic chains are linked uniformly through the same acceptor site of ubiquitin (e.g., K48-linked chains), whereas heterotypic chains are linked through multiple sites and can be further classified as either mixed or branched. Mixed chains are composed of ubiquitin subunits that are modified on only a single acceptor site and are thus topologically equivalent to homotypic chains. By contrast, branched chains contain at least one ubiquitin subunit that is modified concurrently on more than one site, resulting in a branched or “forked” structure. While the structures and functions of many homotypic chains are well understood (Komander and Rape, 2012; Akutsu et al., 2016; Swatek and Komander, 2016), those of heterotypic chains are understood in much less detail. Nevertheless, considerable progress has been made in elucidating the architectures and physiological functions of branched chains over the past 5–6 years (French et al., 2021; Kolla, et al., 2022). They act as powerful degradation signals to ensure the timely removal of regulatory and misfolded proteins from cells, and they activate signaling pathways through degradation-independent mechanisms. In this mini review, we explore the molecular mechanisms that underlie the assembly and disassembly of branched chains, highlighting new findings regarding the chemically-induced formation of branched chains by small molecules and the cleavage of branched chains by UCH37. Although there have been many recent reports suggestive of branched chain architectures, we focus here on cases in which branched structures have been clearly demonstrated through the use of mass spectrometry and/or careful biochemical analysis.

## Assembly of branched chains

Ubiquitin signals are attached to substrates by the concerted actions of E1 ubiquitin-activating enzymes, E2 ubiquitin-conjugating enzymes, and E3 ubiquitin ligases. The human genome encodes ∼600 E3s (Li et al., 2008) with varying substrate specificities, enabling the modification of thousands of targets with ubiquitin monomers or chains of various configurations. Major classes of eukaryotic E3s include those containing a homologous to E6AP C-terminus (HECT), really interesting new gene (RING)/U-box, or RING-between-RING (RBR) domain (Zheng and Shabek, 2017; Lorenz, 2018; Walden and Rittinger, 2018). HECT and RBR E3s form a transient thioester intermediate with ubiquitin before transferring ubiquitin to the substrate and are generally thought to specify the type of ubiquitin chain linkage. By contrast, canonical RING/U-box E3s promote the direct transfer of ubiquitin from E2 to substrate and depend on the E2 to determine the type of chain linkage (Wright et al., 2016). In addition, several new classes of E3s with distinct mechanisms have recently been identified (Horn-Ghetko and Schulman, 2022), although the chain forming activities of these E3s have not been characterized in detail. While the mechanisms of homotypic chain formation are well established for many E2s and E3s (Deol et al., 2019), the events that underlie the formation of branched chains are not nearly as well understood.

Given that members of all major classes of E3s have the ability to form branched polymers (Table 1), the mechanisms of chain branching are expected to be intricate and diverse. The general mechanisms of assembly can be grouped into four different categories. Members of the HECT and RBR classes of E3s, including NleL, UBE3C, Parkin, HECTD1, and WWP1, have the ability to form branched chains of various configurations in cooperation with a single E2 (Wang et al., 2006; Hospenthal et al., 2013; French et al., 2017; Swatek et al., 2019; Deol et al., 2020a; Chen et al., 2021; Harris et al., 2021). The anaphase-promoting complex (APC/C), a multisubunit RING E3, cooperates with two different E2s in a sequential fashion to produce branched K11/K48 polymers (Meyer and Rape, 2014; Yau et al., 2017). A similar mechanism has been described for the RING E3 cIAP1, which synthesizes branched chains containing K48/K63 and K11/K48 linkages in a manner that depends on the activities of UBE2D and UBE2N-UBE2V (Akizuki et al., 2023). Collaboration between pairs of E3s with distinct chain linkage preferences is another common mechanism of branched chain formation. For example, the HECT E3s ITCH and UBR5 collaborate to form branched K48/K63 chains on TXNIP (Figure 1B), whereas Ufd2, a U-box E3, cooperates with the HECT E3 Ufd4 to form branched K29/K48 chains on substrates of the ubiquitin fusion degradation pathway (Liu et al., 2017; Ohtake et al., 2018). Finally, it has been reported that yeast Ubc1 and its mammalian orthologue UBE2K promote the assembly of branched K48/K63 chains (Pluska et al., 2021), indicating that some E2s have an innate chain branching activity.

**TABLE 1:** Summary of E2s and E3s with reported chain branching activities.

E2s and E3s	Linkage type	*In vitro*/*in vivo*	Substrates	Effect on substrates	References
**APC/C + UBE2C + UBE2S**	K11/K48	*In vitro* and *in vivo*	Cyclin A, NEK2A, histone H2B	Proteasomal degradation	Meyer and Rape (2014), Yau et al. (2017), Oh et al. (2020)
**cIAP1 + UBE2D + UBE2N/UBE2V**	K11/K48/K63	*In vitro* and *in vivo*	cIAP1, ER-α	Proteasomal degradation (chemically induced)	Akizuki et al. (2023)
**CRL2** ^**VHL**^ **+ TRIP12**	K29/K48	*In vitro* and *in vivo*	BRD4	Proteasomal degradation (PROTAC-dependent)	Kaiho-Soma et al. (2021)
**HECTD1**	K29/K48	*In vitro*	Unknown	Unknown	Harris et al. (2021)
**IpaH9.8**	K6/K48	*In vitro*	Unknown	Unknown	Valkevich et al. (2014)
**ITCH + UBR5**	K48/K63	*In vitro* and *in vivo*	TXNIP	Proteasomal degradation	Ohtake et al. (2018)
**Parkin**	K6/K48	*In vitro*	Unknown	Unknown	Swatek et al. (2019), Deol et al. (2020a)
**TRAF6 + HUWE1**	K48/K63	*In vitro* and *in vivo*	TRAF6	Inhibition of CYLD cleavage	Ohtake et al. (2016)
**NlEL**	K6/K48	*In vitro*	Unknown	Unknown	Hospenthal et al. (2013), Valkevich et al. (2014)
**Ubc1/UBE2K**	K48/K63	*In vitro*	Unknown	Unknown	Pluska et al. (2021)
**UBE2D + multiple RING E3s**	Multiple types	*In vitro*	Luciferase, troponin I	Inhibition of proteasomal degradation	Kim et al. (2007), Swatek et al. (2019)
**UBE3C**	K29/K48	*In vitro* and *in vivo*	VPS34	Proteasomal degradation	Wang et al. (2006), Chen et al. (2021)
**Ufd4 + Ufd2**	K29/K48	*In vitro* and *in vivo*	Ub-V-GFP	Proteasomal degradation	Liu et al. (2017)
**UBR5 + K11-specific E2/E3**	K11/K48	*In vitro* and *in vivo*	73Q-HTT	Proteasomal degradation	Yau et al. (2017)
**WWP1**	K11/K48/K63	*In vitro*	WBP2	Unknown	French et al. (2017)

Regardless of the E2s and E3s involved, the initiation of chain branching requires the selection of the appropriate branch point linkage and location. Specificity for chain branching, as opposed to elongation, occurs through the recognition of an unbranched chain and the selection of an internal ubiquitin within the chain by the branching E2 or E3. For example, Ufd2 recognizes homotypic K29 chains formed by Ufd4 through its two N-terminal loops to nucleate the formation of branched K29/K48 chains (Liu et al., 2017). Likewise, HUWE1 recognizes homotypic K63 linkages assembled by TRAF6 through its UIM and UBA domains to produce branched K48/K63 chains (Ohtake et al., 2016). For E3s that have both a chain initiating and chain branching activity such as HECTD1 and WWP1, the mechanisms of branching are less clear. A noncovalent ubiquitin-binding site intrinsic to the E3 is likely responsible for orienting the internal acceptor ubiquitin within the chain to facilitate branching. It is unclear how such a site would be regulated to ensure that branching takes place at the appropriate time and location. Furthermore, in almost all cases where branching has been reported, it is unclear how the branch point ubiquitin is selected and how many branch points are present. New methods and approaches are needed to decipher the architectures of branched chains and to fully elucidate their mechanisms of synthesis.

Recent work has shown that branched chains are synthesized in response to small molecules that trigger the forced degradation of otherwise stable substrates. The VHL-based and BRD4-directed proteolysis-targeting chimera (PROTAC) MZ1 induces the formation of branched K29/K48 chains on BRD4. In this case, the mechanism of branched chain synthesis involves the PROTAC-dependent recruitment of CRL2^VHL^, a K48-specific cullin-RING E3, and TRIP12, a K29-specific HECT E3, to BRD4 (Kaiho-Soma et al., 2021) (Figure 1C). Similarly, the small molecule “degrader” LCL-161, which targets the inhibitor of apoptosis protein (IAP) family of E3s for self-ubiquitylation and degradation, triggers the formation of branched chains containing K11, K48, and K63 linkages. In this context, chain branching requires the activities of the UBE2D family of E2s and the K63-specific UBE2N-UBE2V E2 complex. An IAP-based PROTAC that targets the estrogen receptor (ER-α) also induces the formation of K48 and K63 chains in a UBE2N-dependent manner, suggesting a common mechanism of branched chain formation shared by IAP degraders and IAP-based PROTACs (Akizuki et al., 2023). Interestingly, in the case of branched K29/K48 chains assembled by CRL2^VHL^ and TRIP12, TRIP12 appears to have a dual role in attaching K29 linkages to K48 chains synthesized by CRL2^VHL^ and in further stimulating the K48 chain synthesis activity of CRL2^VHL^ (Akizuki et al., 2023). This feedforward mechanism of branching results in the formation of complex chain architectures that are likely to have a direct impact on the degradation efficiency of BRD4 and other related PROTAC substrates (Figure 1C).

Given the therapeutic potential of PROTACs and other small molecule degraders, the mechanisms of chain branching on targeted substrates are of considerable interest. It is interesting to note that TRIP12 is not involved in the degradation of HIF-1α, an endogenous substrate of CRL2^VHL^, suggesting a unique mechanism of degradation by PROTACs that depends on the formation of branched K29/K48 chains (Akizuki et al., 2023). It has been proposed that the requirement for TRIP12 and branched K29/K48 chains can be explained by the fact that PROTAC-directed substrates are normally stable proteins that have not been evolutionarily optimized for degradation. Presumably, such suboptimal substrates require a different type of ubiquitin signal in order to be properly recognized and processed by the degradation machinery. Other classes of suboptimal or “hard-to-degrade” substrates that have been demonstrated or suggested to be degraded in a manner that depends on branched chains include aggregation-prone misfolded proteins, membrane-associated proteins, and proteins that are part of larger macromolecular complexes (Liu et al., 2017; Yau et al., 2017; Samant et al., 2018; Leto et al., 2019; Chen et al., 2021). The quality control pathways that stimulate chain branching E2s and E3s to recognize and modify these substrates in order to trigger their proteasomal degradation are not fully understood.

## Disassembly of branched chains

The disassembly of branched chains and all other types of ubiquitin polymers requires the actions of deubiquitylases (DUBs), which are organized into seven different evolutionarily conserved families. The ∼100 DUBs encoded by the human genome are diverse in terms of their functions and biochemical activities. DUBs that target substrates modified with ubiquitin chains can cleave from the distal end of the chain (an exo-DUB activity), internally within the chain (an endo-DUB activity), or at the point of attachment between the substrate and the proximal ubiquitin to release an intact chain (an *en bloc* chain cleavage activity). Furthermore, while many DUBs show remarkable selectivity for the types of chain linkages they hydrolyze, others, especially those of the ubiquitin-specific protease (USP) family, are nonspecific and cleave chains of multiple linkage types and configurations (Mevissen and Komander, 2017; Clague et al., 2019). While several DUBs have been shown to cleave homotypic linkages within branched chains, as discussed in more detail below, UCH37/UCHL5 is currently the only known DUB to preferentially cleave at the branch point linkage between ubiquitin subunits within a chain.

UCH37 is a proteasome-associated DUB of the ubiquitin C-terminal hydrolase (UCH) family that was originally characterized as a K48-specific isopeptidase with exo-DUB activity that rescues substrates from degradation (Lam et al., 1997; de Poot et al., 2017). Recent studies, however, have shown that UCH37 has an unprecedented ability to selectively cleave at the branch point of a chain containing a K48 linkage (Deol et al., 2020b; Song et al., 2021). UCH37 efficiently debranches K6/K48, K11/K48, and K48/K63 chains (Deol et al., 2020b; Song et al., 2021), whereas it shows significantly weaker or no activity at all against homotypic and mixed chains containing K48 linkages (Yao et al., 2006; Bett et al., 2015; Lu et al., 2017; Deol et al., 2020b; Song et al., 2021). The debranching activity of UCH37 appears to promote rather than inhibit degradation of substrates by the proteasome (Deol et al., 2020b; Du et al., 2022), and it has been suggested that the function of debranching is, in part, to clear ubiquitin chains with complex higher order topologies from the proteasome, thus facilitating further rounds of substrate engagement and processing (Song et al., 2021). Notably, the debranching activity of UCH37 is greatly stimulated by its association with the proteasomal subunit RPN13 (Figure 1D), one of the three main ubiquitin receptors of the proteasome 19 S regulatory particle (Deol et al., 2020b).

How is the specificity of UCH37 for K48 branch points achieved? Although the precise mechanism is unknown, three key observations have shed light on the molecular basis of branch point cleavage. First, the UCH37/RPN13 complex binds to both distal ubiquitin subunits that emanate from the branch point of a K6/K48 chain to increase the affinity of the enzyme for the branch point (Song et al., 2021) (Figure 1D). The fact that the affinity of branched K11/K48 and K48/K63 chains for UCH37/RPN13 is similar to that of homotypic chains, however, indicates that binding specificity alone cannot fully explain the selectivity for branch point cleavage (Song et al., 2021). Second, RPN13 binds to UCH37 on a surface that includes the active-site crossover loop (Sahtoe et al., 2015; VanderLinden et al., 2015), a feature of all UCH DUBs that plays a crucial role in regulating access to the canonical active site of the enzyme. Through mechanisms that are not yet clear, the interaction between RPN13 and the UCH37 active-site crossover loop restricts the cleavage of homotypic chains and promotes the cleavage of branched chains (Song et al., 2021), thereby increasing the debranching specificity of the complex. Third, and perhaps most surprisingly, it has recently been demonstrated that UCH37 has a K48 chain binding site on the backside of the enzyme (at a site distant from the canonical UCH active site) that is required for chain debranching (Du et al., 2022). Molecular docking and chemical crosslinking experiments suggest that both branched and homotypic K48 chains sample the backside of the enzyme in multiple orientations, but only the branched chain configuration results in a catalytically competent arrangement in which the K48 linkage is accessible to the catalytic cysteine of UCH37 (Du et al., 2022).

Several other DUBs have been shown to cleave homotypic linkages within branched chains and thus have the ability to trim branched polymers in addition to cleaving homotypic chains. USP30, a DUB that preferentially disassembles homotypic K6 linkages (Cunningham et al., 2015; Gersch et al., 2017), also cleaves branched K6/K48 chains *in vitro* (Deol et al., 2020b). This activity is consistent with the role of USP30 in antagonizing ubiquitylation events mediated by Parkin (Bingol et al., 2014; Liang et al., 2015; Gersch et al., 2017; Ordureau et al., 2020), an E3 that assembles chains containing K6, K11, K48, and K63 linkages (Ordureau et al., 2014; Swatek et al., 2019). The ovarian tumor protease (OTU) family DUB Cezanne cleaves K11 linkages in the context of branched K11/K48 chains *in vitro* and has been shown to deubiquitylate APC/C substrates *in vivo* (Mevissen et al., 2016; Bonacci et al., 2018). TRABID/ZRANB1, another OTU family DUB, cleaves K29 linkages within branched K29/K48 chains attached to the E3 HECTD1, suggesting a DUB chain editing function (Harris et al., 2021). An editing function may allow DUBs that target substrates modified with branched chains to alter the landscape of the chains in a way that has a range of different functional outcomes for the target protein. It will be of interest, for example, to determine if chain debranching has the ability rescue certain substrates from degradation or to regulate protein activity through degradation-independent mechanisms.

## Concluding remarks

Although considerable progress has been made in revealing the architectures and functions of branched ubiquitin chains in recent years, there are still many questions that remain unanswered. In most cases, the higher-order configurations of these chains, including the number of branch points and the length of the branches, are not well understood. The mechanisms that govern the selection of the branch point ubiquitin(s) and those that stimulate the branching of an otherwise unbranched polymer are also poorly understood. While the ubiquitin chain restriction (UbiCRest) method has proven to be somewhat useful in deciphering the order of ubiquitin linkages within a branched chain (Hospenthal et al., 2015; Harris et al., 2021; Akizuki et al., 2023), new techniques and approaches will ultimately be needed to reveal the precise architectures of branched chains and to fully elucidate their mechanisms of synthesis. It will be of great interest to determine if chain branching is a general property shared by many E2s and E3s or if branching is limited to a dedicated set of “E4”-like enzymes. Of note, HUWE1, UBR4, UBR5, and TRIP12 have been reported to have a branching activity in more than one context (Ohtake et al., 2016; Yau et al., 2017; Ohtake et al., 2018; Khan et al., 2021; Akizuki et al., 2023), perhaps indicating a division of labor between linkage-specific and architecture-specific E3s.

The discovery of UCH37 as the first DUB with remarkable specificity for cleaving at the branch point of a ubiquitin chain has paved the way for new and exciting areas of research. While other debranching enzymes have not been reported as of yet, it is noteworthy that branched chains have been detected in *S. cerevisiae* (Liu et al., 2017; Pluska et al., 2021), which lacks a UCH37 orthologue. Despite progress in understanding how branch point specificity is achieved by UCH37 at the molecular level, the precise role of RPN13 in contributing to this process remains elusive. Based on existing data, it seems likely that RPN13 stabilizes a conformation of UCH37 that is conducive to branched chain binding. It is also possible that RPN13 provides a binding site for the non-K48-linked ubiquitin that emanates from the branch point to stabilize a catalytic architecture favorable for debranching (Du et al., 2022). Structural work will almost certainly be needed in order to distinguish between these possibilities. Finally, it may be possible to harness the unique specificity of UCH37 for debranching in order to identify new targets of branched chains and to explore the biological functions of these enigmatic signals. Given the recent explosion in the number of reports of chain branching E2s and E3s, it seems probable that many such targets, both endogenous and therapeutic, will be discovered in the coming years.
